# Cross-national and historical variation in relative age effects in professional football

**DOI:** 10.1371/journal.pone.0354928

**Published:** 2026-10-01

**Authors:** Thomas Ulrich Grund

**Affiliations:** Institute of Sociology, RWTH Aachen University, Aachen, Germany; Charles Sturt University, AUSTRALIA

## Abstract

The relative age effect (RAE) — the overrepresentation of players born early in the selection year — is one of the most robust findings in sports science, yet how much it varies across countries and over time remains surprisingly underexplored. Drawing on birth-date data for 49,578 male professional footballers across 44 national systems and five birth cohorts (1985–2009), this study describes three patterns that have not previously been examined together. RAE magnitude varies enormously across national football systems, ranging from close to zero to birth-quarter gaps exceeding 40 percentage points. It has also increased across successive birth cohorts: players born in 2005–2009 show birth-quarter imbalances roughly 9 percentage points wider than those born in 1985–1989. Common macro-level indicators — national footballing strength, gross domestic product (GDP) per capita, and population — show at most weak and inconsistent associations with cross-national differences in RAE and do not account for the historical increase once country and cohort are held fixed. Together, these findings provide a comparative benchmark for understanding cross-national and historical variation in relative age effects.

## 1. Introduction

Elite footballers are disproportionately born early in the selection year. Since Barnsley, Thompson, and Barnsley’s discovery of this pattern in junior ice hockey [1], relative age effects (RAEs) have been documented across dozens of sports and national contexts, including football [2], ice hockey, and basketball, and confirmed by successive meta-analyses [3,4]. Within any nominally same-aged cohort, relatively older children may enjoy physical, cognitive, and behavioural advantages over their younger peers [5,6]. These initial differences are not, however, the whole of the phenomenon. They can be amplified through selection and through the secondary benefits that follow it: players identified as more promising may receive better coaching, more playing opportunities, stronger competition, and other developmental advantages that accumulate over time, and there is good reason to treat relative age and biological maturity as distinct rather than interchangeable constructs [7]. Among adult professional footballers, players born in the first quarter of the selection year typically outnumber those born in the fourth quarter by ratios exceeding two to one [8,9].

The resulting literature has concentrated primarily on establishing that RAEs exist and on identifying the mechanisms — maturational advantage, coach bias, dropout among late-born players — that plausibly sustain them at the individual level [3,10]. This work has also examined how RAE magnitude varies with moderators such as competition level and talent-identification intensity [11,12].

Football provides an especially informative setting for these questions because it combines highly standardised age-group selection with substantial institutional variation across countries. Existing theories implicitly treat RAE as a near-universal consequence of grouping children by birth date within a selection year, with variation across settings read as secondary noise around a common phenomenon. Two questions that follow directly from that assumption remain underexplored: how much RAE actually differs between national football systems, and whether it has changed across historical time. Existing studies typically examine a single country or a small set of countries at a single point in time, and large-scale comparative evidence remains scarce [8,13–15]. A sufficiently large and comparable dataset can answer both questions directly, without first having to decide between competing explanations for why RAE occurs at all.

This study provides one of the largest comparative analyses of professional footballers to examine cross-national and historical variation in RAE simultaneously. Using birth-date data on 49,578 male professional footballers across 44 national systems and five birth cohorts (1985–2009), it reports substantial cross-national heterogeneity in RAE magnitude, a systematic increase in RAE across successive birth cohorts, and at most weak and inconsistent associations between common macro-level indicators — national footballing strength, gross domestic product (GDP) per capita, and population — and either pattern. If RAE were a fixed by-product of grouping children by age, its magnitude should not range from close to zero to more than forty percentage points across countries applying essentially the same selection rule; documenting that it does is itself a finding that constrains how the phenomenon can be theorised, independent of any particular explanation for the variation. The paper does not attempt to determine which mechanisms produce these patterns; it treats them as a benchmark for future explanatory work and considers candidate explanations only briefly, in the Discussion.

## 2. Background

### 2.1 Relative age effects in sport

Decades of research have established the basic contours of the relative age effect. Within age-grouped selection systems, relatively older children may initially demonstrate physical, cognitive, and behavioural advantages that coaches reliably — if mistakenly — interpret as indicators of superior athletic talent [10], but RAE is best understood as a developmental process in which these initial differences interact with selection decisions and with the opportunities subsequently afforded to selected players [7]. Children identified early as talented tend to receive more coaching, more deliberate practice, and richer competitive exposure, which can widen the gap between early- and late-born children regardless of the initial talent distribution [16]. These dynamics are further compounded by coach-expectation effects, differences in confidence and self-efficacy, and self-selection dropout among late-born children who anticipate exclusion [3]. Meta-analytic reviews confirm that RAEs are pervasive across sports, age groups, and national contexts, and that performance level is among the strongest moderators of effect magnitude: effects tend to be largest where selection is most consequential [3,4].

Considerably less attention has been paid to describing how RAE magnitude compares across national systems, or how it has moved over historical time. A notable exception is a study of RAE across international soccer World Cups, which documented shifts in birth-quarter imbalances over time within a small set of elite national teams [13]. Another examined RAE across clubs and countries within Europe’s Big Five leagues, concluding that no club or country was systematically immune to the bias, though without a historical dimension or coverage beyond Europe’s top leagues [17]; the present study extends this focus on cross-national and temporal variation to a much larger and more representative sample of professional club players spanning 44 national systems.

Building on this literature, this study addresses three questions: how much RAEs differ across national football systems; how RAEs have changed across birth cohorts; and to what extent these patterns are associated with common macro-level indicators — national footballing strength, economic development, and population size.

## 3. Data and methods

### 3.1 Data sources and analytical sample

The analytical dataset combines publicly available player, football performance, and macroeconomic data. The underlying player records originate from Transfermarkt and were obtained through a publicly available Kaggle dataset that provides structured extracts of Transfermarkt data, including player birth dates, nationality, club affiliations, transfers, market values, appearances, injuries, and national-team history. Historical national team strength was measured using the publicly available International Football Elo Ratings (1872–2025) dataset available on Kaggle. Macroeconomic indicators were retrieved directly from the World Bank World Development Indicators via the World Bank API and include annual gross domestic product (GDP), GDP per capita, and total population. Transfermarkt has become a standard source for research on professional football because it provides comprehensive, regularly updated player histories spanning leagues and countries, including the birth-date and nationality fields on which the present analysis depends [18,19].

The player database initially contained approximately 93,000 professional footballers. The analytical sample was constructed in several stages. First, players were restricted to those with complete information on birth year, birth month, and nationality. Because nationality serves as a proxy for the national football environment in which players completed their formative development, it was then matched to the corresponding national football system for the construction of the formative Elo measures described below; players for whom this match could not be established were excluded. Players born outside the study period (1985–2009) were also removed. Countries whose youth football systems use a registration cut-off other than 1 January were not excluded; instead, birth quarters were defined relative to each country’s own cut-off date (Section 3.2); England and Wales use a 1 September cut-off rather than the calendar-year system common elsewhere, so players from these countries were assigned to Q1–Q4 relative to 1 September rather than 1 January.

The sample comprises male professional players represented in the Transfermarkt database rather than players drawn from a single harmonised competition tier. Consequently, the precise level of professional competition represented is not identical across national systems: in some countries the recorded player pool is dominated by the top domestic division, whereas in others it extends further down the professional pyramid or includes a larger share of players employed abroad. This design is appropriate for the study’s aim of characterising national professional-player pools, but it means that cross-national differences may partly reflect differences in the composition and coverage of professional football across countries rather than differences in selection practice alone. To reduce instability arising from sparse coverage, country-cohort cells with fewer than 25 players were excluded, and the share of players competing in a Big Five top-tier league and the share competing domestically are included as covariates to capture at least some of this compositional variation.

National football strength was measured using annual Elo ratings. For each player, the formative football environment was defined as ages 12–16, corresponding to the period during which players typically enter elite youth development pathways [20,21]. Annual national team Elo ratings across these years were averaged to produce a formative Elo score, and players were required to have Elo observations for at least three formative years. Players without sufficient Elo coverage were excluded from the analysis.

Players were assigned to birth quarters relative to the youth registration cut-off used in their national football system. For almost all national football systems in the sample, the youth registration year follows the calendar year (1 January). The only exceptions were England and Wales, where youth age groups are organised using a 1 September cut-off. For these two countries, birth quarters were therefore recoded relative to the September cut-off rather than the calendar year.

The player-level data were then aggregated into country-by-birth-cohort cells using five five-year birth cohorts (1985–1989, 1990–1994, 1995–1999, 2000–2004, and 2005–2009). Country-cohort cells containing fewer than 25 players were excluded to ensure stable estimates of relative age effects. For each remaining cell, the relative age effect was measured as the difference between the proportion of players born in the first and fourth birth quarters (Q1 share − Q4 share), and the shares born in each of the four birth quarters were retained for the descriptive and sensitivity analyses reported below. Each country-cohort was additionally linked to its average formative Elo rating, the proportion of players competing in a top-tier league within Europe’s Big Five leagues, the proportion of players competing domestically, and annual GDP, GDP per capita, and population measured at the midpoint of the cohort’s formative period. Macroeconomic indicators were winsorised at the 1% level before analysis.

Two sample stages should be distinguished. The player-level cohort panel — all players with complete birth-date and nationality information and sufficient formative Elo coverage — contained 49,578 professional footballers. After excluding country-cohort cells with fewer than 25 players, the country-cohort analyses comprised 173 cells from 44 national football systems, representing 47,723 of those players. All models, figures, and quarter shares reported below are based on the 173 retained cells. Descriptive statistics are presented in Table 1. All data processing, integration, and analysis scripts are available in the accompanying Open Science Framework repository.

**Table 1:** Descriptive statistics for the country-cohort analytical sample.

Variable	N	Mean	SD	Min	Max
Countries	44				
Country-cohort observations	173				
Players in cohort panel	49,578				
Players in retained country-cohort cells	47,723				
Birth cohorts	5				
Players per country-cohort	173	275.855	430.892	25.000	3265.000
RAE gap (Q1 − Q4 share)	173	0.170	0.096	−0.156	0.407
Q1 share	173	0.339	0.062	0.144	0.473
Q2 share	173	0.269	0.049	0.074	0.409
Q3 share	173	0.222	0.047	0.100	0.360
Q4 share	173	0.170	0.050	0.030	0.352
Log Q1/Q4 odds ratio	173	0.699	0.422	−0.571	2.269
Formative Elo rating	173	1804.689	142.519	1480.126	2119.821
Formative Elo rating (z-score)	173	0.000	1.000	−2.277	2.211
Share in Big Five top tier	173	0.037	0.053	0.000	0.387
Share domestic players	173	0.251	0.199	0.000	0.769
Log GDP per capita, formative midpoint	173	9.647	1.176	6.728	11.393

Note. The cohort panel comprises all players with complete birth-date, nationality, and formative Elo information; the country-cohort analyses are based on the 47,723 of these players who fall in cells retained after the minimum cell-size restriction. All remaining rows describe the 173 retained country-cohort cells. Formative Elo is the mean national Elo rating across ages 12–16 for each cohort. Quarter shares are country-cohort means.

### 3.2 Measuring the relative age effect

The primary dependent variable is the RAE gap: Q1 share minus Q4 share within each country-cohort cell. Positive values indicate greater representation of players born early rather than late in the selection year. This measure treats equal Q1 and Q4 representation as the descriptive reference point. Population births, however, may exhibit seasonal variation across countries and historical periods, so a zero gap need not correspond exactly to the birth distribution that would be expected in the absence of selection. Country- and cohort-specific population birth distributions were not available in sufficiently harmonised form for the 44 national systems and the full historical period examined here, and this limitation is considered explicitly in Section 5.

The log Q1/Q4 odds ratio is used as an alternative specification with desirable distributional properties. Because both measures focus on the endpoints of the birth-year distribution, I additionally examine the full Q1–Q4 distribution and estimate a sensitivity model using the difference between the share born in the first half of the selection year (Q1 + Q2) and the share born in the second half (Q3 + Q4). Substantive conclusions are consistent across these operationalisations.

### 3.3 Country-cohort design

The analytical unit is the country-cohort — a five-year birth cohort within a national football system. This design corrects a temporal misalignment inherent in cross-sectional approaches: RAE in a professional player pool reflects selection decisions made during adolescence, ten to fifteen years before observation. A player born in 1990 and observed in 2015 was channelled into or out of elite development around 2002–2006; the relevant developmental environment is the national football context of those years, not 2015. For each player born in year b, the formative window spans ages 12–16 (years b + 12 to b + 16), and mean national Elo across that window is averaged within country-cohort cells. Country fixed effects absorb stable, time-invariant national characteristics. Within-country cohort-to-cohort variation in formative Elo captures changes in competitive standing, which allows the association between footballing strength and RAE to be assessed separately from stable between-country differences.

### 3.4 Covariates

The primary explanatory variable is formative-period Elo, standardised for interpretability [22]. Because Elo ratings are derived from senior national-team results, formative Elo is best understood as an indirect proxy for a country’s football standing at the time, not a direct measure of the quality or structure of its youth development system; results involving this variable are interpreted accordingly throughout. Additional covariates assess whether other macro-level indicators are associated with residual variation: log GDP per capita and log population at the cohort’s formative midpoint; the share of players in a Big Five top-tier league (elite pathway access) [23] — measured as current senior appearances at the cohort’s formative midpoint, not peak career or youth-academy affiliation — and the share of players competing domestically (pathway insularity). Because these football-structure variables reflect realised playing quality as much as pathway access, their associations with RAE are interpreted descriptively rather than as evidence of an institutional mechanism. These are deliberately the most commonly available macro-level indicators rather than direct measures of developmental organisation: academy licensing dates, youth academies, and youth registration rates would test an institutional account more directly, but are not available in comparable form across 44 countries and five decades. All covariates are measured at the formative midpoint to align with the developmental period relevant for RAE.

Because total GDP is mechanically related to GDP per capita and population, it is not included simultaneously with these two variables in the preferred specification. As a sensitivity analysis, the fixed-effects models were re-estimated entering total GDP, GDP per capita, and population in alternative specifications (S3 Table).

### 3.5 Analytical strategy

I estimated six OLS models (Table 2). Although the RAE gap is bounded between −1 and 1, observed values cluster well within the interior of that range (Table 1), so a linear specification is unlikely to distort inference. Model 1 is pooled with no fixed effects, relating formative Elo alone to the RAE gap. Model 2 retains the pooled specification but adds log GDP per capita, log population, the share of players in Big Five top-tier leagues, and the share competing domestically. Model 3 introduces country and birth-cohort fixed effects, separating within-country dynamics from stable national differences, with formative Elo as the sole predictor. Model 4 combines the fixed-effects specification with the full set of macroeconomic and football-structure covariates. Model 5 uses the log Q1/Q4 odds ratio as an alternative dependent variable. Model 6 uses the difference between the combined Q1 + Q2 and Q3 + Q4 shares, thereby incorporating information from all four birth quarters. All models are weighted by players per cell, so that country-cohort observations built from more players carry proportionately more influence over the estimates; standard errors are clustered by country. Cohorts are defined in five-year windows to balance temporal resolution against cell stability — narrower windows would generate many more country-cohort cells below the minimum player threshold, at the cost of noisier estimates.

**Table 2:** Country-cohort regression models of relative age imbalance.

	Model 1 Pooled	Model 2 Pooled + Full	Model 3 FE	Model 4 FE + Full	Model 5 Log Q1/Q4	Model 6 All-quarter DV
Formative Elo (z-score)	0.024** (0.008)	0.015 (0.015)	0.000 (0.015)	0.010 (0.020)	−0.018 (0.062)	−0.011 (0.025)
Log GDP per capita		0.004 (0.013)		−0.029 (0.028)		
Log population		0.007 (0.013)		0.125 (0.193)		
Share Big Five top tier		−0.202* (0.095)		−0.419** (0.138)		
Share domestic players		0.122** (0.044)		−0.075 (0.071)		
Cohort 1990–1994			−0.005 (0.013)	0.023 (0.021)	−0.008 (0.060)	0.003 (0.027)
Cohort 1995–1999			0.019 (0.017)	0.065* (0.028)	0.098 (0.076)	0.030 (0.029)
Cohort 2000–2004			0.060*** (0.017)	0.099*** (0.028)	0.253*** (0.073)	0.088** (0.030)
Cohort 2005–2009			0.089*** (0.026)	0.147*** (0.035)	0.411*** (0.116)	0.148*** (0.037)
Observations	173	173	173	173	173	173
R²	0.079	0.164	0.714	0.761	0.720	0.691
Adjusted R²	0.073	0.139	0.604	0.657	0.612	0.571
Country FE	No	No	Yes	Yes	Yes	Yes
Cohort FE	No	No	Yes	Yes	Yes	Yes

Note. Country-clustered HC1 standard errors in parentheses. Baseline cohort: 1985–1989. Models are weighted by players per country-cohort cell. Models 1–4 use the Q1 − Q4 share gap as the outcome; Model 5 uses the log Q1/Q4 odds ratio; Model 6 uses the (Q1 + Q2) − (Q3 + Q4) share gap. ***p < .001; **p < .01; *p < .05; †p < .10.

To assess sensitivity to the macroeconomic specification, I additionally estimated fixed-effects models entering total GDP, GDP per capita, and population separately, alongside the preferred specification containing GDP per capita and population together. These results are reported in S3 Table.

## 4. Results

### 4.1 Strong but uneven: The global distribution of RAE

Across 173 country-cohort observations, the mean RAE gap was 17.0 percentage points (SD = 9.6), ranging from −15.6 to 40.7 percentage points (Table 1). Aggregating the 47,723 players across these cells, 35.4% were born in Q1, 27.1% in Q2, 21.8% in Q3, and 15.7% in Q4. Representation therefore declined progressively across the birth year rather than differing only between its endpoints. Fig 1 displays the distribution of RAE gaps across country-cohort observations.

**Fig 1:**
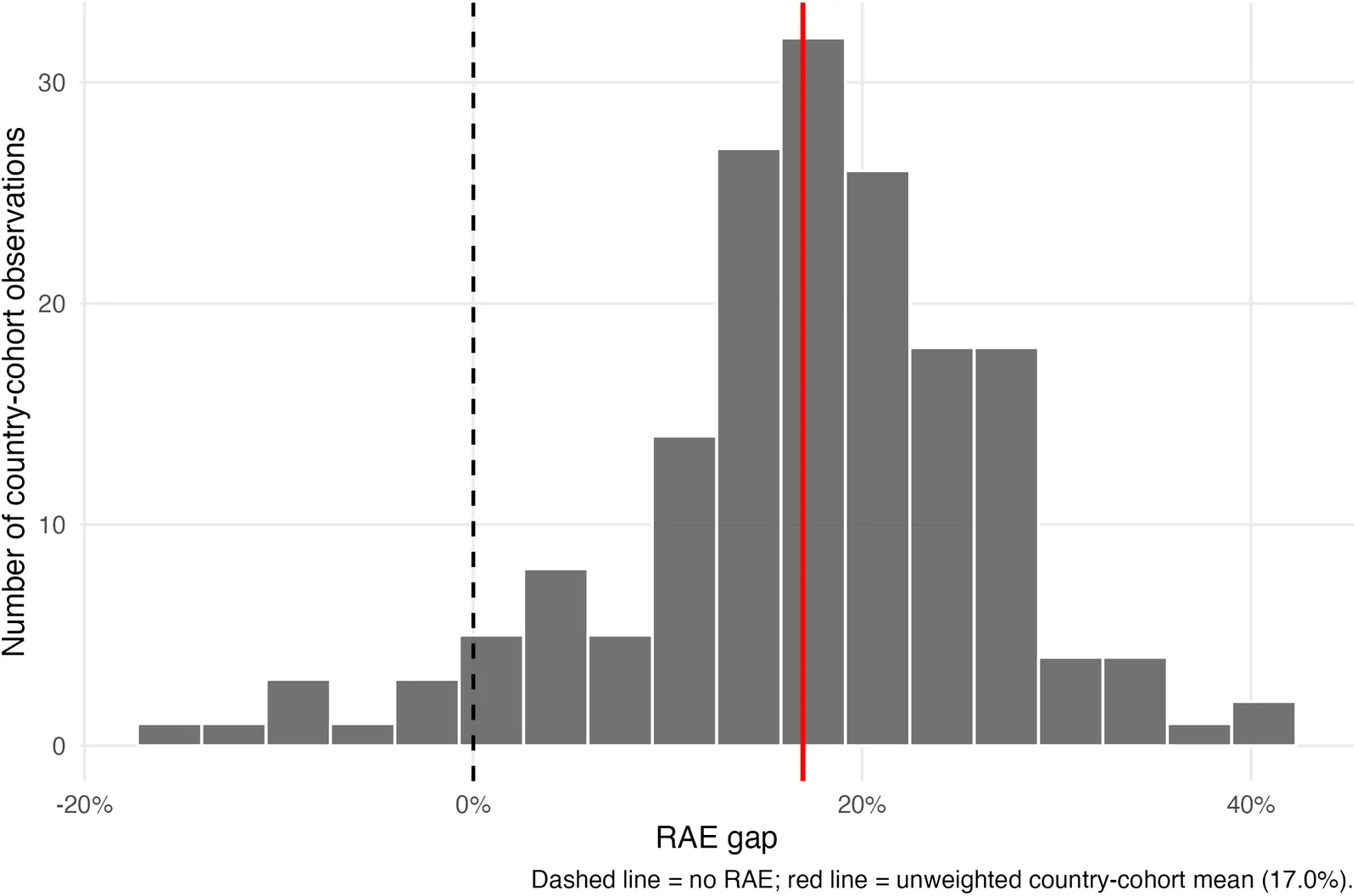
Distribution of birth-quarter imbalance (Q1 − Q4 share) across country-cohort observations. Dashed vertical line = no RAE; red line = unweighted country-cohort mean (17.0%).

The distribution is wide, positively skewed, and spans a striking range: some country-cohorts cluster near zero, while others exceed 40 percentage points. RAE magnitude, in other words, is not a fixed property of age-grouped developmental sport that operates the same way wherever football is played; it varies substantially, and at present the sources of that variation are poorly understood.

### 4.2 Historical intensification across birth cohorts

Fig 2 shows that each successive generation of professional footballers exhibits a wider birth-quarter gap than the one before. Grey lines show individual national trajectories, while the orange line shows the player-weighted average across country-cohort observations. Birth-quarter imbalances increase modestly among players born during the 1990s and more sharply among cohorts born after 2000. Consistent with this descriptive pattern, the fixed-effects model estimates that the RAE gap was 8.9 percentage points larger for the 2005–2009 birth cohort than for the 1985–1989 cohort (SE = 2.6 percentage points, p < .001; Model 3, Table 2). Because the models include country fixed effects, the cohort coefficients capture within-country changes over time rather than stable differences between national football systems. In other words, the same national systems that produced relatively modest birth-quarter imbalances for earlier generations tended to produce substantially larger imbalances for later ones.

**Fig 2:**
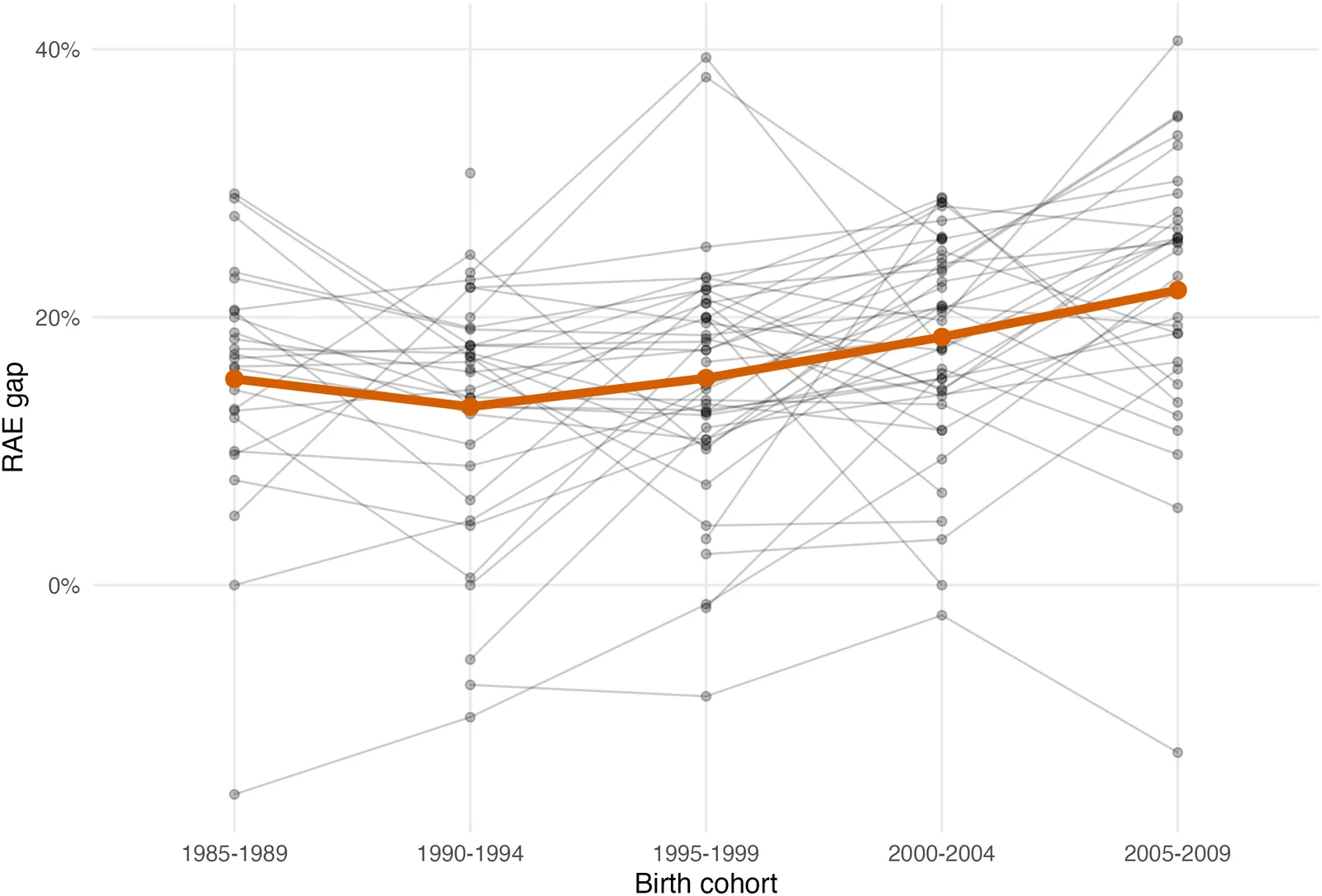
Country-level RAE trajectories across birth cohorts. Grey lines represent individual national systems; the orange line shows the player-weighted average across country-cohort observations.

The historical increase is not confined to the contrast between Q1 and Q4. Fig 3 displays the complete birth-quarter distribution across cohorts. Q1 representation rises across the more recent cohorts while Q4 representation declines; Q2 representation also increases slightly and Q3 representation falls, so that the whole distribution becomes progressively more skewed towards earlier birth quarters. Consistent with this descriptive pattern, the alternative model comparing the first half of the selection year (Q1 + Q2) with the second half (Q3 + Q4) shows a substantial increase across cohorts: in Model 6, the 2005–2009 cohort difference is approximately 14.8 percentage points relative to 1985–1989 (p < .001). The corresponding cohort shares are reported in S2 Table.

**Fig 3:**
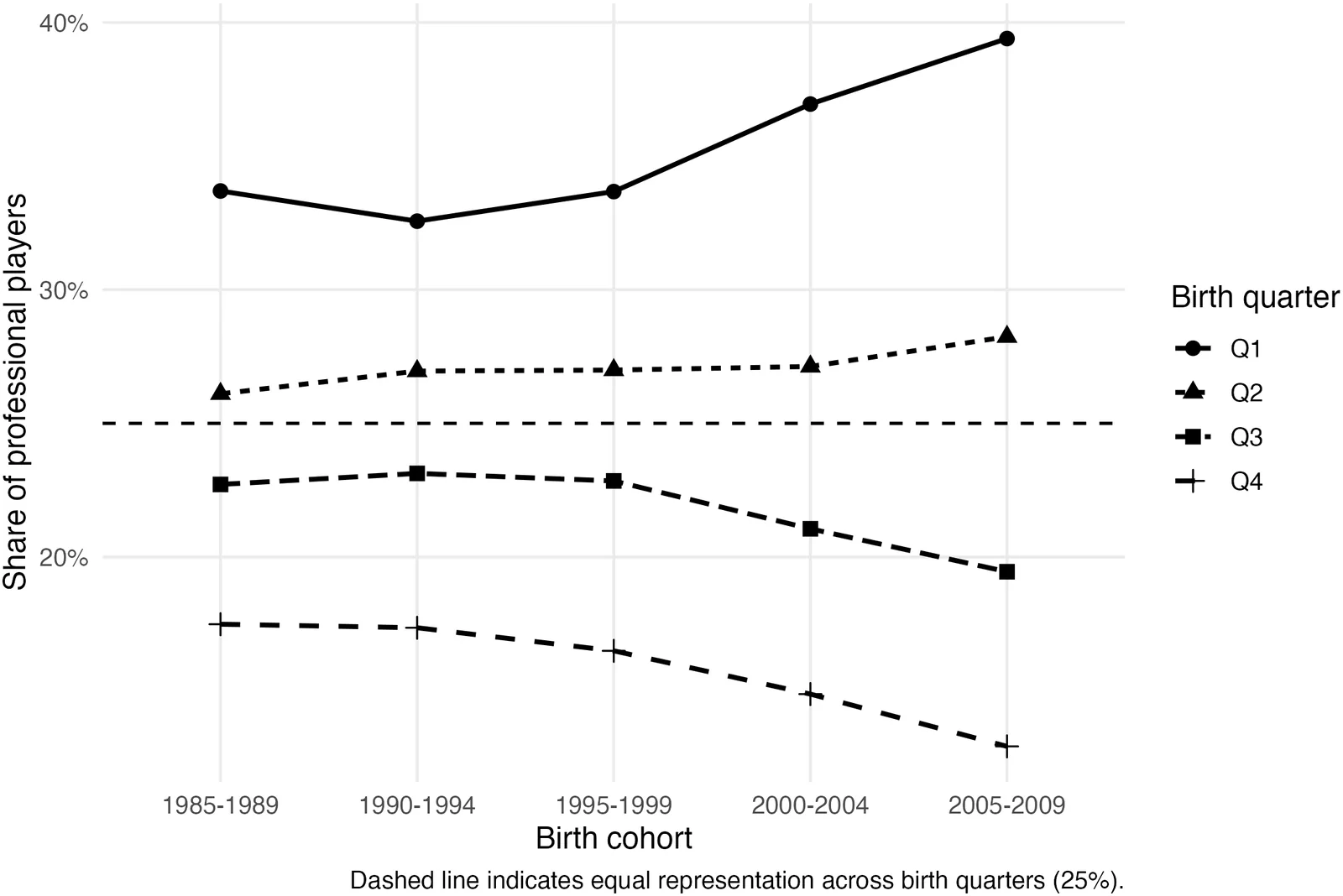
Share of professional players born in each birth quarter, by birth cohort. Dashed line indicates equal representation across birth quarters (25%).

Although most countries exhibit increasing trajectories, there is substantial heterogeneity in their magnitude. Twenty-six of the 35 countries with at least three observed cohorts show positive slopes, with a median increase of 2.6 percentage points per five-year birth cohort, while nine display flat or declining trajectories (S1 Fig and S2 Fig). A player-level multilevel logistic model nested within national football systems reproduces the same positive cohort trend (S1 Table), indicating that the pattern is not an artefact of aggregating players into country-cohort observations.

### 4.3 Cross-national heterogeneity in RAE

Fig 4 shows country-level mean RAE gaps ranked from lowest to highest, making the cross-national range concrete. Japan, Senegal, Cameroon, and Ghana sit at the lower extreme, their elite player populations showing little or no systematic birth-quarter sorting, consistent with earlier single-country evidence that some national youth football systems exhibit minimal relative age effects [14]. At the upper extreme, Türkiye, Guinea, Romania, Paraguay, Morocco, and Qatar show the largest mean gaps. This range — from close to zero to extreme — raises the question of why countries whose players are subject to broadly similar developmental processes exhibit such different levels of RAE. What accounts for the range is not identified by the data analysed here and is considered briefly in the Discussion. Bubble size in Fig 4 is proportional to the number of players underlying each estimate; countries at either extreme with small bubbles are estimated from fewer players, and their rankings should be read with correspondingly more caution.

**Fig 4:**
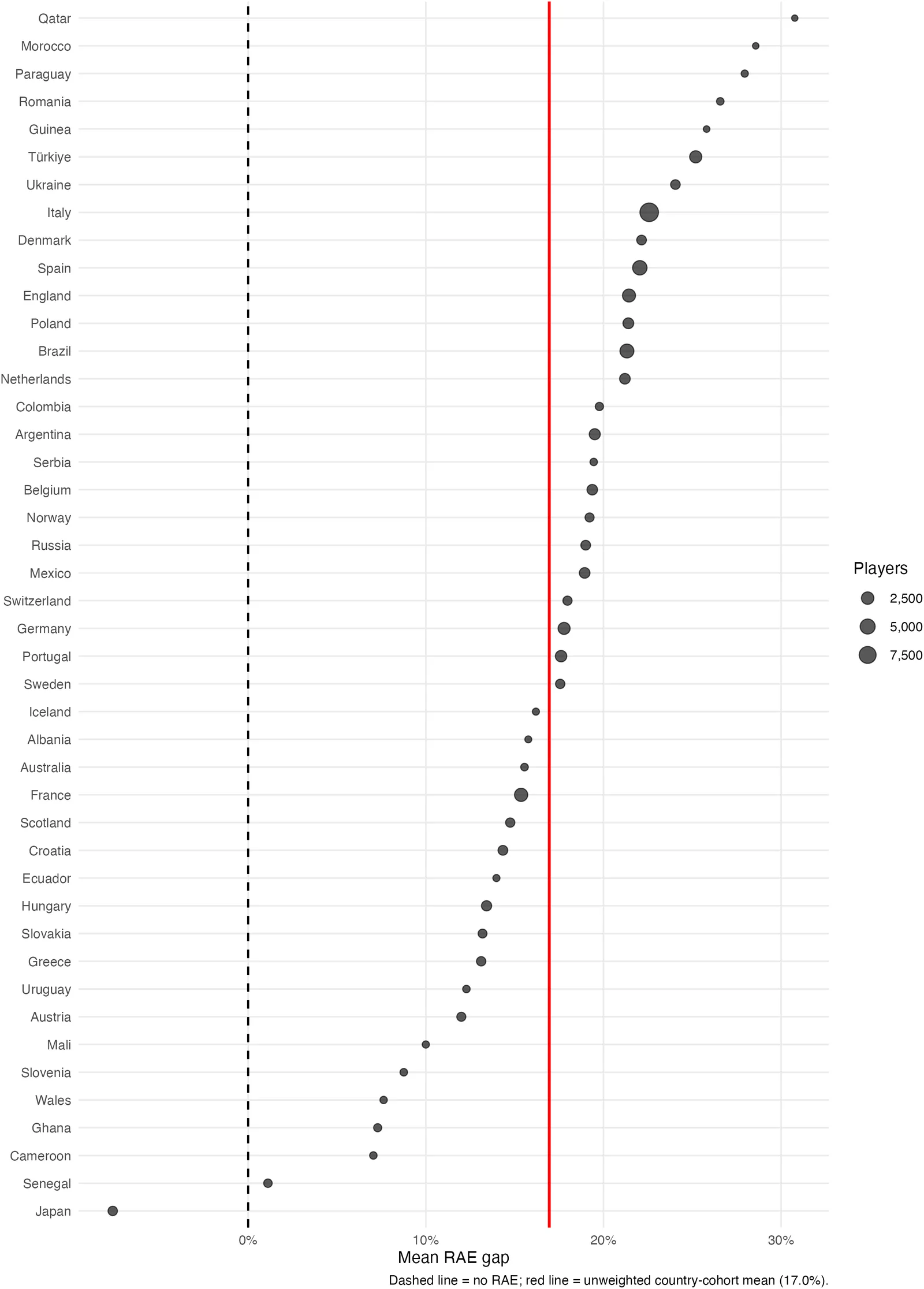
Mean RAE gap by country, ordered lowest to highest. Bubble size proportional to total players. Dashed line = no RAE; red line = unweighted country-cohort mean RAE gap (17.0%). Countries represented by fewer players (smaller bubbles) carry more sampling uncertainty.

### 4.4 Macro-level indicators explain little of the variation in RAE

The regression results in Table 2 examine whether the observed cross-national differences in RAEs are associated with commonly proposed macro-level explanations. In the pooled cross-national comparison (Model 1), stronger football systems are associated with larger RAEs: formative Elo has a positive and statistically significant association with the RAE gap (β = 0.024, SE = 0.008, p < .01). A one-standard-deviation increase in formative Elo (approximately 140 rating points) corresponds to an approximately 2.4-percentage-point larger RAE gap. Although statistically reliable, the association is substantively modest, with formative Elo alone explaining only a small share of the cross-national variation (R^2^ = .08).

Adding GDP per capita, population, Big Five representation, and domestic share attenuates this association (Model 2). Formative Elo is no longer statistically significant (β = 0.015, SE = 0.015), while GDP per capita (β = 0.004, SE = 0.013) and population (β = 0.007, SE = 0.013) show no significant associations with RAEs. Two football-structure covariates reach conventional levels of statistical significance in this pooled specification: the share of players competing in Big Five top-tier leagues is negatively associated with the RAE gap (β = −0.202, SE = 0.095, p < .05), and the share of players competing domestically is positively associated with it (β = 0.122, SE = 0.044, p < .01). Together, however, these variables increase the explained variance only modestly (R^2^ = .16).

Once country and birth-cohort fixed effects are introduced (Model 3), the picture changes. Within-country changes in formative national strength are unrelated to changes in RAEs across cohorts (β = 0.000, SE = 0.015). Countries that became stronger footballing nations during the study period did not exhibit correspondingly larger RAEs in the cohorts that came of age during that strengthening. Adding the macroeconomic and football-structure variables (Model 4) does not alter this conclusion. Formative Elo remains unrelated to RAEs (β = 0.010, SE = 0.020), while GDP per capita (β = −0.029, SE = 0.028) and population (β = 0.125, SE = 0.193) continue to show no significant associations. The share of players competing in Big Five leagues remains negatively associated with RAEs (β = −0.419, SE = 0.138, p < .01), whereas the domestic-player share is not statistically significant once country and cohort differences are held fixed (β = −0.075, SE = 0.071). This conclusion is unchanged using either the log Q1/Q4 outcome (Model 5: β = −0.018, SE = 0.062) or the all-quarter Q1 + Q2 versus Q3 + Q4 measure (Model 6: β = −0.011, SE = 0.025).

Taken together, these results indicate that the positive cross-national association between footballing strength and RAE largely reflects stable differences between countries rather than changes within countries over time. Standard macroeconomic indicators explain little of the observed variation in RAEs, both across countries and across cohorts. While countries with greater representation in Europe’s Big Five leagues tend to exhibit smaller RAEs, this association does not account for the pronounced cohort escalation documented in Section 4.2. Alternative macroeconomic specifications entering total GDP, GDP per capita, and population separately produce the same substantive conclusions (S3 Table). The factors driving the substantial increase in RAEs over successive birth cohorts therefore remain largely unexplained by the macro-level characteristics examined here.

## 5. Discussion

The most striking finding is not that relative age effects exist, but that their magnitude varies dramatically across football systems, ranging from virtually zero to more than 40 percentage points. The developmental asymmetries that initially accompany relative age are broadly shared across age-grouped sporting systems, but their consequences depend on subsequent selection, opportunity, coaching, competition, and other cumulative processes, and it is these downstream processes that are most likely to differ across the countries examined here. Much previous research has treated variation in effect size as secondary once the existence of RAE has been established. The present findings instead shift attention from asking whether RAE exists to understanding why it becomes so much stronger in some developmental environments than in others. This cross-national heterogeneity is only part of the story because the same systems have also changed markedly over time. The descriptive comparisons across countries (Section 4.3) characterise how much RAE differs between national systems; the country-cohort fixed-effects models (Section 4.4) characterise how RAE has changed within these same systems over successive cohorts. Together they describe two distinct dimensions of RAE variation — between countries, and within countries over time — rather than a single, unified estimate of the phenomenon.

The historical pattern is perhaps the most surprising result. Relative age effects have been documented for more than four decades, during which awareness among researchers, coaches, and governing bodies has increased substantially [9,20]. If awareness alone were sufficient to reduce selection bias, one might expect RAEs to stabilise or even decline. Instead, the opposite pattern emerges. Successive birth cohorts display increasingly pronounced RAEs, indicating that the processes generating selection advantages for relatively older players have intensified over time, despite growing recognition of the phenomenon. Importantly, the sensitivity analyses show that this historical pattern is not an artefact of contrasting only the first and fourth quarters: the full Q1–Q4 distribution becomes increasingly skewed towards earlier birth quarters (Fig 3), and the Q1 + Q2 versus Q3 + Q4 specification reproduces the cohort increase (Model 6, Table 2).

The macro-level analyses help narrow the range of plausible explanations. Footballing strength, measured by formative Elo, is associated with larger RAEs in simple cross-national comparisons, but this relationship weakens substantially after accounting for broader country characteristics and disappears entirely once stable country differences are taken into account. Likewise, GDP per capita and population show little relationship with the magnitude of RAEs in the preferred specification, and sensitivity analyses entering total GDP, GDP per capita, and population separately lead to the same substantive conclusion (S3 Table). The only consistent macro-level correlate is the share of players competing in Europe’s Big Five leagues, which is negatively associated with RAE intensity. This finding suggests that countries with stronger integration into elite European club football may exhibit somewhat weaker selection biases, although the mechanisms underlying this association remain unclear. The domestic-player share, by contrast, is not consistently associated with RAE once country and cohort differences are taken into account. More importantly, none of the macro-level indicators considered here explains the pronounced escalation of RAEs across successive birth cohorts.

The key drivers of relative age effects are therefore unlikely to lie in broad differences in national wealth or footballing success, and more likely to lie in characteristics of talent development systems themselves, including when players are identified, how selection decisions are made, the extent to which developmental pathways remain open to later developers, and the incentives facing clubs and academies — organisational features of professional academies that prior work has shown to vary considerably even among elite European clubs [24,25]. The present data cannot directly evaluate these institutional mechanisms, but the large unexplained differences between countries and the substantial historical increase indicate that they are likely to be more consequential than the macro-level characteristics examined here.

Previous work has proposed several candidate moderators of this institutional variation, including the intensity of competition for playing time and roster places [14], the turnover and organisation of academy and national-youth talent-promotion programmes [25], coaches’ selection criteria and their awareness of relative age when identifying talent [20], and broader talent-identification practices that shape how and when players are selected [21], although evidence linking these specific mechanisms to cross-national differences in RAE magnitude remains fragmented [26].

Future research should therefore move beyond broad national indicators and collect comparable information on developmental institutions, including academy entry ages, scouting practices, competition structures, selection procedures, and opportunities for re-entry after deselection. Such data would allow researchers to explain not only why countries differ in the magnitude of their RAEs, but also why these effects have strengthened over successive generations. Extending this comparative perspective to women’s football, where relative age effects are also documented but have not been benchmarked cross-nationally or historically to the same degree [27], as comparable longitudinal data become available, represents a natural next step.

Several features of the data warrant caution. A first limitation concerns the reference distribution used to identify relative age imbalance. The analyses compare observed birth-quarter shares with equal representation across the selection year, but population births themselves can be seasonal, and the extent of that seasonality may vary across countries and historical periods. Harmonised country- and cohort-specific monthly birth distributions were not available across the full sample of 44 national systems and five birth cohorts. Consequently, some portion of the observed cross-national or temporal differences could reflect underlying variation in population birth seasonality rather than selection processes. The pronounced and increasingly monotonic Q1–Q4 gradient observed in the professional-player sample is consistent with an RAE, but future comparative work should ideally benchmark player distributions against contemporaneous population births.

A second limitation concerns coverage and comparability. The analytical sample covers 44 national systems with sufficient Transfermarkt coverage and complete Elo data and is weighted towards European and larger football nations. Consequently, the estimated cohort trends may not generalise equally to smaller or less well-documented systems. The professional level represented in Transfermarkt is also not perfectly standardised across national systems, so some cross-national differences may reflect variation in the composition and coverage of professional player pools rather than differences in selection practice. Formative Elo is derived from senior international performance and therefore provides only an indirect proxy for youth development quality. Likewise, although the share of players competing in Big Five leagues is consistently associated with RAE intensity, it should not be interpreted causally because it is likely to capture a broader set of institutional and developmental characteristics that remain unobserved.

Finally, the sample reflects survivorship. Only players who reached sufficient professional prominence to appear in Transfermarkt are observed, implying that the estimated RAEs are likely conservative relative to the selection processes operating earlier in development [28–30]. In addition, the youngest cohort (2005–2009) comprises players who were still between sixteen and twenty years old at the time of data collection. As these careers unfold, the observed cohort trend may change, although the increasing pattern documented across earlier cohorts suggests that, if anything, the eventual differences are more likely to widen than diminish. Notably, the historical increase does not depend on this youngest, still-maturing cohort: Table 2 shows a statistically significant rise already by the 2000–2004 cohort (β = 0.060, p < .001, Model 3) relative to the 1985–1989 baseline, before the 2005–2009 cohort most exposed to survivorship concerns is even considered. The historical intensification therefore remains evident when the analysis is restricted to the first four cohorts (1985–2004), suggesting that survivorship bias in the most recent cohort, while a genuine limitation, is unlikely to be the sole driver of the overall pattern. Other features of the data could also contribute to the observed trend. Transfermarkt’s coverage has expanded over the period studied, and if earlier cohorts are reconstructed retrospectively while recent cohorts are captured contemporaneously, differential coverage could inflate the apparent increase; similarly, the broader professionalisation and growth of the global player pool since the 1980s could raise measured RAE even if underlying selection bias were constant. I cannot rule out these possibilities with the present data, though neither implies that the cross-national heterogeneity documented in Section 4.3 is an artefact, since that comparison holds the observation window fixed across countries. Finally, the analysis is restricted to men’s football because Transfermarkt’s coverage of women’s football is substantially less complete over this study period, which precluded constructing a comparably harmonised country-cohort sample.

## 6. Conclusion

Relative age effects are neither universal nor historically fixed. Using birth-date data on 49,578 professional footballers, of whom 47,723 fall within the 173 country-cohort observations analysed across 44 national systems and five birth cohorts (1985–2009), this study documents substantial and previously underappreciated variation in RAE across both countries and generations. RAE magnitude ranges from close to zero to birth-quarter gaps exceeding 40 percentage points across national football systems, while the average birth-quarter imbalance increases by approximately 9 percentage points between the 1985–1989 and 2005–2009 birth cohorts. The historical increase is also evident across the complete birth-quarter distribution rather than being confined to the Q1–Q4 contrast. Although stronger footballing nations exhibit larger RAEs in simple cross-national comparisons, this association disappears once stable country differences are taken into account. Likewise, GDP per capita and population explain little of the observed variation, and this conclusion is unchanged across alternative macroeconomic specifications. Countries with greater representation in Europe’s Big Five leagues tend to exhibit somewhat weaker RAEs, but this relationship does not account for the pronounced historical increase documented here.

Rather than providing a definitive explanation for these patterns, this study establishes them as a comparative benchmark for future research. Comparative benchmarks are a prerequisite for explanation. Before theories can explain why developmental systems differ, robust evidence that they do is needed first. The findings suggest that institutional features of talent identification and player development, including the timing of selection, academy structures, scouting practices, and opportunities for later entry, are more promising candidates than broad national characteristics. Testing these explanations, however, will require comparable cross-national data on developmental institutions that are not yet available.

More broadly, this study suggests that relative age effects should be understood as a property of developmental systems rather than simply an individual-level selection bias. Once attention shifts from documenting the existence of RAE to explaining variation in its magnitude across countries and over time, new comparative questions emerge. By providing a reproducible benchmark for both cross-national and historical variation in professional football, this study lays the empirical foundation for understanding why some developmental systems amplify relative age effects far more than others and, ultimately, how these inequalities might be reduced.

## Supporting information

S1 Fig Distribution of country-specific RAE cohort slopes. Positive values indicate increasing RAE across cohorts. Countries with at least three observed cohorts are included.
(TIF)

S2 Fig Country-specific change in RAE across birth cohorts. Countries are ranked from the steepest increase to the steepest decrease. Bubble size is proportional to total players.
(TIF)

S1 Table Player-level multilevel logistic models predicting Q1 rather than Q4 birth quarter. Players are nested within national football systems; models include formative Elo as a control.
(DOCX)

S2 Table Birth-quarter distributions by birth cohort. Reports the share of players born in each of the four birth quarters, the Q1 − Q4 gap, and the (Q1 + Q2) − (Q3 + Q4) gap for each five-year birth cohort.
(DOCX)

S3 Table Sensitivity of macro-level results to alternative economic specifications. Fixed-effects models entering total GDP, GDP per capita, and population separately, alongside the preferred specification containing GDP per capita and population together.
(DOCX)

### Declaration of generative AI use

The author used Claude (Anthropic) during the editing and revision of this manuscript to assist with restructuring, clarity, and language. The research design, data collection, analyses, interpretation of results, and conclusions were developed and verified solely by the author. All AI-assisted revisions were reviewed and approved by the author, who takes full responsibility for the accuracy, originality, and integrity of the manuscript, including all references and citations.
