# Supplementary material for: Cross-national and historical variation in relative age effects in professional football
Source: PLoS One. 2026 Oct 1;21(10):e0354928. doi: 10.1371/journal.pone.0354928 (PMC13630218; doi:10.1371/journal.pone.0354928)
Supplement: Supplementary file 1 [file pone.0354928.s003.docx]

# **Player-Level Robustness Check**

As a further robustness check on the cohort trend reported in Section 4.2, I estimated multilevel logistic regression models predicting whether a professional player was born in Q1 rather than Q4, with players nested within national football systems (S1 Table). Because the binary outcome compares only the first and fourth birth quarters, players born in Q2 and Q3 are excluded from these models; the complementary all-quarter evidence is reported in S2 Table and in Model 6 of Table 2. Model 1 includes birth-cohort indicators, a country random intercept, and formative Elo as a control. Relative to the 1985–1989 birth cohort, the odds of a Q1 birth increase across successive cohorts: 1990–1994 (OR = 1.02, ns), 1995–1999 (OR = 1.11, p < .05), 2000–2004 (OR = 1.31, p < .001), and 2005–2009 (OR = 1.52, p < .001). Model 2 replaces the cohort indicators with a single linear cohort trend and allows that trend to vary across countries through a random slope. Each successive five-year birth cohort is associated with an 11.3% increase in the odds of a Q1 rather than Q4 birth (OR = 1.113, p < .001). Formative Elo is positively associated with the probability of a Q1 birth in both models (OR = 1.07–1.08, p < .01). Together, these player-level analyses confirm that the historical intensification reported in Section 4.2 is not an artefact of aggregating players into country-cohort observations.

**S1 Table. *Player-level multilevel logistic models predicting Q1 rather than Q4 birth quarter.***

|  | **Model 1: Random intercept** | **Model 2: Random intercept + slope** |
| --- | --- | --- |
| Intercept | 1.715*** (0.106) | 1.454*** (0.103) |
| Cohort 1990–1994 | 1.023 (0.054) |  |
| Cohort 1995–1999 | 1.108* (0.057) |  |
| Cohort 2000–2004 | 1.312*** (0.066) |  |
| Cohort 2005–2009 | 1.515*** (0.089) |  |
| Cohort number |  | 1.113*** (0.019) |
| Formative Elo (z-score) | 1.069** (0.026) | 1.077** (0.027) |
| Observations | 25,313 | 25,313 |
| R² marginal | 0.008 | 0.007 |
| R² conditional | 0.024 | 0.024 |
| Cohort indicators | Yes | No |
| Linear cohort trend | No | Yes |
| Formative Elo control | Yes | Yes |
| Country random intercept | Yes | Yes |
| Country random cohort slope | No | Yes |

*Note. Exponentiated coefficients are odds ratios; standard errors in parentheses. Outcome is Q1 versus Q4 birth quarter among professional players. ***p < .001; **p < .01; *p < .05.*
