# Supplementary material for: Cross-national and historical variation in relative age effects in professional football
Source: PLoS One. 2026 Oct 1;21(10):e0354928. doi: 10.1371/journal.pone.0354928 (PMC13630218; doi:10.1371/journal.pone.0354928)
Supplement: Supplementary file 2 [file pone.0354928.s004.docx]

# **Birth-Quarter Distributions Across All Four Quarters**

Because both the RAE gap and the log Q1/Q4 odds ratio use only the endpoints of the birth-year distribution, S2 Table reports, for every five-year birth cohort, the share of players born in each of the four birth quarters across the 173 retained country-cohort cells (47,723 players in total), together with the Q1 − Q4 gap and the (Q1 + Q2) − (Q3 + Q4) gap. Representation declines monotonically from Q1 to Q4 in every cohort, and the gradient steepens over historical time: the Q1 share rises from 33.7% in the 1985–1989 cohort to 39.4% in the 2005–2009 cohort, while the Q4 share falls from 17.5% to 12.9%. The early–late gap based on all four quarters increases from 19.6 to 35.3 percentage points across the same period, closely tracking the pattern obtained from the Q1 − Q4 contrast. These distributions are displayed graphically in Fig 3 and are used as the dependent variable in Model 6 of Table 2.

**S2 Table. *Birth-quarter distributions by birth cohort.***

| **Birth cohort** | **Country-cohort observations** | **Players** | **Q1 share** | **Q2 share** | **Q3 share** | **Q4 share** | **Q1 − Q4 gap** | **(Q1+Q2) − (Q3+Q4) gap** |
| --- | --- | --- | --- | --- | --- | --- | --- | --- |
| 1985–1989 | 25 | 4,455 | 33.7% | 26.1% | 22.7% | 17.5% | 16.2% | 19.6% |
| 1990–1994 | 34 | 9,560 | 32.6% | 27.0% | 23.1% | 17.4% | 15.2% | 19.0% |
| 1995–1999 | 41 | 11,188 | 33.7% | 27.0% | 22.8% | 16.5% | 17.2% | 21.3% |
| 2000–2004 | 42 | 15,250 | 36.9% | 27.1% | 21.1% | 14.9% | 22.1% | 28.1% |
| 2005–2009 | 31 | 7,270 | 39.4% | 28.2% | 19.4% | 12.9% | 26.5% | 35.3% |

*Note. Quarter shares are computed by summing quarter counts across country-cohort cells within each birth cohort and dividing by the corresponding number of players. Birth quarters are defined relative to each national system’s youth registration cut-off.*
