# Supplementary material for: Cross-national and historical variation in relative age effects in professional football
Source: PLoS One. 2026 Oct 1;21(10):e0354928. doi: 10.1371/journal.pone.0354928 (PMC13630218; doi:10.1371/journal.pone.0354928)
Supplement: Supplementary file 3 [file pone.0354928.s005.docx]

# **Sensitivity to Alternative Macroeconomic Specifications**

Total GDP is mechanically related to GDP per capita and population, so entering all three simultaneously is unnecessarily collinear. The preferred specification reported in Table 2 therefore includes GDP per capita and population while omitting total GDP. S3 Table reports fixed-effects models in which each macroeconomic indicator is entered separately, alongside the preferred specification. Across all four specifications the substantive conclusions are identical: none of the macroeconomic indicators is significantly associated with the RAE gap; formative Elo remains unrelated to RAE once country and cohort fixed effects are included; the share of players in Big Five top-tier leagues remains negatively associated with RAE; the domestic-player share remains non-significant; and the cohort coefficients remain large and highly significant, with the 2005–2009 cohort between 12.9 and 15.9 percentage points above the 1985–1989 baseline. Explained variance is essentially unchanged across specifications (R² = 0.758–0.761).

**S3 Table. *Sensitivity of macro-level results to alternative economic specifications.***

|  | **Total GDP** | **GDP per capita** | **Population** | **GDP pc + Population** |
| --- | --- | --- | --- | --- |
| Formative Elo (z-score) | 0.009 (0.018) | 0.009 (0.019) | 0.008 (0.019) | 0.010 (0.020) |
| Log GDP | −0.022 (0.028) |  |  |  |
| Log GDP per capita |  | −0.027 (0.028) |  | −0.029 (0.028) |
| Log population |  |  | 0.113 (0.195) | 0.125 (0.193) |
| Share Big Five top tier | −0.409** (0.127) | −0.411** (0.128) | −0.414** (0.139) | −0.419** (0.138) |
| Share domestic players | −0.081 (0.076) | −0.080 (0.076) | −0.072 (0.073) | −0.075 (0.071) |
| Cohort 1990–1994 | 0.026 (0.021) | 0.027 (0.020) | 0.008 (0.020) | 0.023 (0.021) |
| Cohort 1995–1999 | 0.070** (0.027) | 0.072** (0.026) | 0.042 (0.029) | 0.065* (0.028) |
| Cohort 2000–2004 | 0.108*** (0.026) | 0.109*** (0.026) | 0.081** (0.031) | 0.099*** (0.028) |
| Cohort 2005–2009 | 0.158*** (0.035) | 0.159*** (0.035) | 0.129** (0.040) | 0.147*** (0.035) |
| Observations | 173 | 173 | 173 | 173 |
| R² | 0.758 | 0.759 | 0.758 | 0.761 |
| Adjusted R² | 0.656 | 0.657 | 0.656 | 0.657 |
| Country FE | Yes | Yes | Yes | Yes |
| Cohort FE | Yes | Yes | Yes | Yes |

*Note. Country-clustered HC1 standard errors in parentheses. Dependent variable: country-cohort RAE gap (Q1 − Q4 share). All models include country and birth-cohort fixed effects and are weighted by players per country-cohort cell. Macroeconomic indicators are entered separately except in the final column, which is the preferred specification reported as Model 4 in Table 2. Baseline cohort: 1985–1989. ***p < .001; **p < .01; *p < .05; †p < .10.*
